# Artificial intelligence-based identification of thin-cap fibroatheromas and clinical outcomes: the PECTUS-AI study

**DOI:** 10.1093/eurheartj/ehaf595

**Published:** 2025-09-01

**Authors:** Rick H J A Volleberg, Thijs J Luttikholt, Ruben G A van der Waerden, Pierandrea Cancian, Joske L van der Zande, Xiaojin Gu, Jan-Quinten Mol, Tomasz Roleder, Mathias Prokop, Clara I Sánchez, Bram van Ginneken, Ivana Išgum, Simone Saitta, Jos Thannhauser, Niels van Royen

**Affiliations:** Department of Cardiology, Radboud University Medical Center, PO Box 9101, Nijmegen 6500 HB, the Netherlands; Department of Cardiology, Radboud University Medical Center, PO Box 9101, Nijmegen 6500 HB, the Netherlands; Diagnostic Image Analysis Group, Radboud University Medical Center, Nijmegen, the Netherlands; Department of Cardiology, Radboud University Medical Center, PO Box 9101, Nijmegen 6500 HB, the Netherlands; Diagnostic Image Analysis Group, Radboud University Medical Center, Nijmegen, the Netherlands; Department of Biomedical Engineering and Physics, Amsterdam University Medical Center, Amsterdam, the Netherlands; Quantitative Healthcare Analysis Group, Informatics Institute, University of Amsterdam, Amsterdam, the Netherlands; Department of Cardiology, Radboud University Medical Center, PO Box 9101, Nijmegen 6500 HB, the Netherlands; Diagnostic Image Analysis Group, Radboud University Medical Center, Nijmegen, the Netherlands; Department of Biomedical Engineering and Physics, Amsterdam University Medical Center, Amsterdam, the Netherlands; Quantitative Healthcare Analysis Group, Informatics Institute, University of Amsterdam, Amsterdam, the Netherlands; Department of Cardiology, Radboud University Medical Center, PO Box 9101, Nijmegen 6500 HB, the Netherlands; Faculty of Medicine, Wrocław University of Science and Technology, Wrocław, Poland; Department of Medical Imaging, Radboud University Medical Center, Nijmegen, the Netherlands; Department of Biomedical Engineering and Physics, Amsterdam University Medical Center, Amsterdam, the Netherlands; Quantitative Healthcare Analysis Group, Informatics Institute, University of Amsterdam, Amsterdam, the Netherlands; Diagnostic Image Analysis Group, Radboud University Medical Center, Nijmegen, the Netherlands; Department of Biomedical Engineering and Physics, Amsterdam University Medical Center, Amsterdam, the Netherlands; Quantitative Healthcare Analysis Group, Informatics Institute, University of Amsterdam, Amsterdam, the Netherlands; Department of Radiology and Nuclear Medicine, Amsterdam University Medical Center, Amsterdam, the Netherlands; Department of Biomedical Engineering and Physics, Amsterdam University Medical Center, Amsterdam, the Netherlands; Quantitative Healthcare Analysis Group, Informatics Institute, University of Amsterdam, Amsterdam, the Netherlands; Department of Cardiology, Radboud University Medical Center, PO Box 9101, Nijmegen 6500 HB, the Netherlands; Diagnostic Image Analysis Group, Radboud University Medical Center, Nijmegen, the Netherlands; Department of Cardiology, Radboud University Medical Center, PO Box 9101, Nijmegen 6500 HB, the Netherlands

**Keywords:** Optical coherence tomography, Artificial intelligence, Deep learning, Myocardial infarction, High-risk plaque, Thin-cap fibroatheroma

## Abstract

**Background and Aims:**

Coronary thin-cap fibroatheromas (TCFA) are associated with adverse outcome, but identification of TCFA requires expertise and is highly time-demanding. This study evaluated the utility of artificial intelligence (AI) for TCFA identification in relation to clinical outcome.

**Methods:**

The PECTUS-AI study is a secondary analysis from the prospective observational PECTUS-obs study, in which 438 patients with myocardial infarction underwent optical coherence tomography (OCT) of all fractional flow reserve-negative non-culprit lesions (i.e. target lesions). OCT images were analyzed for the presence of TCFA by an independent core laboratory (CL-TCFA) and OCT-AID, a recently developed and validated AI segmentation algorithm (AI-TCFA). The primary outcome was defined as the composite of death from any cause, non-fatal myocardial infarction or unplanned revascularisation at 2 years (±30 days), excluding procedural and stent-related events.

**Results:**

Among 414 patients, AI-TCFA and CL-TCFA were identified in 143 (34.5%) and 124 (30.0%) patients, respectively. AI-TCFA within the target lesion was significantly associated with the primary outcome [hazard ratio (HR) 1.99, 95% confidence interval (CI) 1.02–3.90, *P* = .04], while the HR for CL-TCFA was non-significant (1.67, 95% CI: .84–3.30, *P* = .14). When evaluating the complete pullback, AI-TCFA showed an even stronger association with the primary outcome (HR 5.50, 95% CI: 1.94–15.62, *P* < .001; negative predictive value 97.6%, 95% CI: 94.0%–99.3%).

**Conclusions:**

AI-based OCT image analysis allows standardized identification of patients at increased risk of adverse cardiovascular outcome, offering an alternative to manual image analysis. Furthermore, AI-assisted evaluation of complete imaged segments results in better prognostic discrimatory value than evaluation of the target lesion only.

## Structured Graphical Abstract

**Structured Graphical Abstract:**
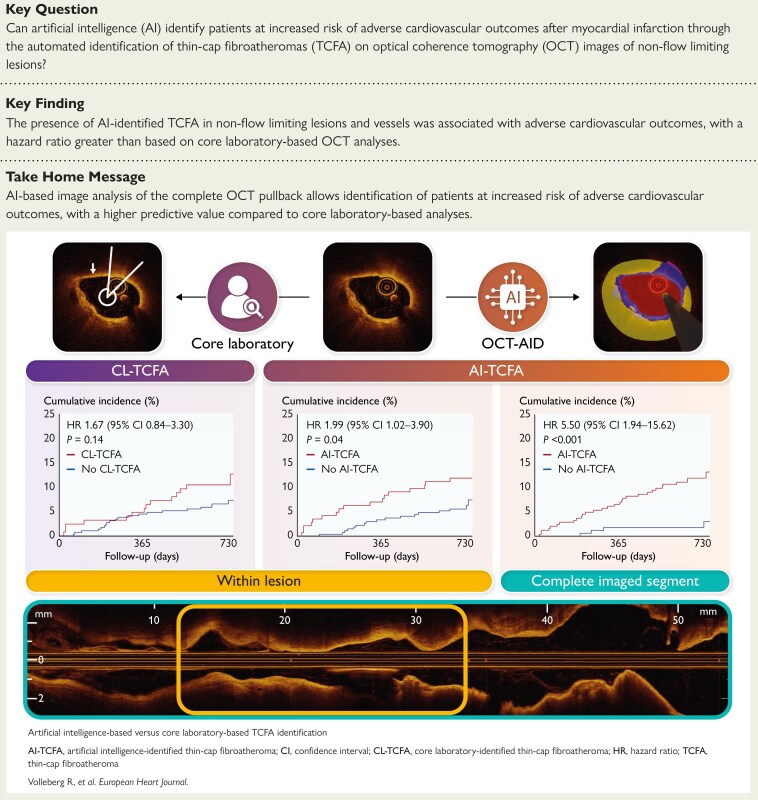

**See the editorial comment for this article ‘Artificial intelligence-based identification of thin-cap fibroatheroma: a new paradigm for risk stratification?’, by J. Lee**  ***et al*****., https://doi.org/10.1093/eurheartj/ehaf662.**

## Introduction

Intracoronary optical coherence tomography (OCT) allows most detailed *in vivo* evaluation of coronary atherosclerosis, including the identification of high-risk plaques. Such plaques are characterized by a large lipid content and a small overlying fibrous cap, which is referred to as a thin-cap fibroatheroma (TCFA).^1^ Multiple prospective observational studies demonstrated that OCT-identified high-risk plaques, when evaluated by experienced core laboratories, are associated with adverse patient-level and lesion-level events, and that they may be a driving force behind coronary events originating from non-flow limiting lesions.^2–5^ However, all these studies were based on offline OCT image analyses by core laboratories, which may reduce the generalizability of the findings to the daily clinical practice where OCT images will be evaluated by operating physicians, who may not have the same experience as core laboratory personnel. Furthermore, high-risk plaque identification is subject to substantial interobserver variability, even among highly experienced core laboratories.^6,7^ Finally, frame-by-frame evaluation of a complete OCT pullback is highly time-demanding, posing a challenge for real-time on-site evaluation. However, immediate on-site image interpretation is essential when pre-emptive treatment of TCFA is to be pursued.^8^ Therefore, automated image interpretation methods that can accurately identify TCFA with an association with adverse clinical outcomes are warranted.

We previously developed and validated OCT-AID, a comprehensive artificial intelligence (AI) model for automated full-vessel segmentation of OCT images.^9^ This AI model is able to accurately quantify plaques, including the fibrous cap thickness, in a standardized manner. However, it remains to be demonstrated whether this algorithm can be used for prognostication.

Therefore, we aimed to evaluate whether our previously proposed AI algorithm can identify TCFA when compared with core lab analyses, and whether AI-detected TCFA is associated with adverse clinical outcome in patients with myocardial infarction and non-culprit plaques.

## Methods

### Study design

The PECTUS-AI study is a pre-planned secondary analysis from the prospective, multicenter, observational PECTUS-obs study (NCT03857971), of which the design and primary results were reported previously.^4,10^ In brief, this study evaluated the association between core lab-identified high-risk plaques in intermediate, non-flow limiting, deferred, non-culprit lesions (i.e. target lesions) and clinical outcome in 438 patients with myocardial infarction. In- and exclusion criteria are listed in Supplementary data online, Method  *S1*. Included patients underwent OCT imaging of all eligible target lesions meeting the criteria of a visually estimated stenosis grade 30%–90% with a fractional flow reserve (FFR) > 0.80. Patients subsequently underwent structured clinical follow-up at 1 and 2 years (±30 days) after inclusion through telephone contact. The study was approved by the institutional review boards and/or medical ethics committee of each participating centre and was conducted in accordance with the 1964 Declaration of Helsinki and its later amendments. Signed informed consent was obtained from all participants.

### OCT image acquisition and core laboratory evaluation

OCT images were acquired using the Dragonfly Optis imaging catheter (Abbott Vascular, Illinois, CA, USA). An independent OCT core laboratory performed blinded OCT image interpretation with the use of the angiogram as reference for localisation of the target lesion(s) on each OCT pullback. OCT image analyses were conducted in accordance with contemporary consensus documents,^11,12^ as described in detail elsewhere.^4^ Core laboratory-identified TCFA (CL-TCFA) was defined as a lipid plaque with a lipid arc ≥90° and a minimum fibrous cap thickness <65 µm at the frame-level. Patients with at least one CL-TCFA were considered CL-TCFA-positive, irrespective of the number of included lesions per patient.

### AI-based OCT image analysis

AI-based OCT image evaluation was performed on the region(s) that were previously identified by the OCT core laboratory as the target lesion(s) and on the complete imaged segment using two previously developed algorithms.^9,13^

#### AI-based plaque segmentation

We built on our previously developed OCT-AID algorithm for multiclass segmentation of OCT pullbacks.^9^ OCT-AID performs pixelwise labelling of each OCT frame, distinguishing among ten classes: background, guidewire artefact, lumen, intima, media, lipid, calcium, side branch, plaque rupture and thrombus. This study introduces an updated version based on nnU-Net version 2,^14^ which integrates a residual encoder U-Net within the previously described self-configuring framework. Details on the algorithm are described in Supplementary data online, Method  *S2*.

The total dataset consisted of 475 pullbacks, of which 207 (43.6%) had manually defined pixelwise reference annotations in a selected set of frames (*n* = 3026) as described previously.^9^ The remaining 268 (56.4%) pullbacks had no reference annotations. We performed stratified 10-fold cross-validation on the frames with reference annotations, ensuring data separation across folds on a patient level. Dataset stratification across folds also maintained a balanced class distribution, with an absolute difference ≤5.0% per class. For each fold, an OCT-AID model instance was trained on 90% of this dataset and evaluated on the left out 10% (see Supplementary data online, Method  *S3*). Model predictions were used to evaluate algorithm performance on a frame-level and pixel-level (see Supplementary data online, Method  *S4*).

The pullbacks without reference annotations were divided into 10 subsets of approximately equal size. Each of these 10 subsets was segmented by a trained OCT-AID model instance selected to ensure separation between the training and test data on the patient level and thereby preventing data leakage.

#### AI-based artefact detection and frame/lesion exclusion

A previously developed deep learning-based algorithm was used for the identification of A-lines containing severe attenuation artefacts caused by blood or gas bubbles, which impair image interpretation.^13^ Frames were excluded from further analyses if >25% of A-lines were affected by severe artefacts. Subsequently, lesions that retained <50% of their frames were excluded from further analyses. The same was done for pullbacks for the pullback-level analyses.

#### AI-based TCFA identification

After exclusion of frames affected by artefacts, the automatically segmented frames were included in the final analysis and were used for quantification of lipid plaques, including the calculation of the lipid arc and minimum fibrous cap thickness as described before.^9^ The latter was calculated as the shortest distance between segmented lumen an lipid pixels measured from the centroid of the lumen, ignoring directly neighbouring pixels. An AI-identified TCFA (AI-TCFA) was defined as a lipid plaque with a lipid arc ≥90° and a minimum fibrous cap thickness <65 µm in at least 3 out of 10 consecutive frames. Patients with at least one AI-TCFA were considered AI-TCFA-positive, irrespective of the number of included lesions per patient.

### Clinical outcome

The primary outcome was defined as the composite of death from any cause, non-fatal myocardial infarction or unplanned revascularisation at 2-year follow-up (±30 days). Periprocedural and stent failure-related events and myocardial infarction not clearly attributable to a specific coronary segment were not considered for the current analysis. Secondary outcomes included the individual components of the primary outcome. All potential events were evaluated by a central, independent event adjudication committee consisting of two members who were blinded to the OCT images and OCT image evaluation results. Events were adjudicated with the use of health records obtained from participating centres, general practitioners and/or other treating physicians, and by comparing index and event angiograms.

### Statistical analysis

Categorical variables are summarized as absolute values (percentages) and compared using the Chi-squared test or Fisher's exact test. Normally distributed continuous variables are presented as means ± standard deviation and analyzed using the Student’s independent samples *t*-test. Non-normally distributed variables are provided as median (interquartile range [IQR]) and evaluated using the Mann–Whitney *U* test. The correspondence between AI-TCFA and CL-TCFA is presented using 2 × 2 contingency tables and was evaluated using Cohen’s Kappa. Values are reported with 95% confidence intervals (CI).

Clinical outcomes were evaluated on a patient level according to the presence or absence of AI-TCFA or CL-TCFA in any target lesion, irrespective of the number of included lesions per patient. This analysis is based on the rationale that the potential risk associated with the presence of high-risk plaques is not restricted to the identified TCFA site. Instead, it is considered a marker of patient-level vulnerability. Second, we evaluated the presence of AI-TCFA within the complete imaged segment. Time-to-event data for the primary outcome is presented using Kaplan-Meier curves and was evaluated using the log-rank test. The hazard ratio (HR) for the clinical outcomes associated with the presence of at least one TCFA was estimated using univariable Cox proportional hazard models. The discriminatory value of AI-TCFA (within the target lesion and within the complete imaged segment) and CL-TCFA (within the target lesion) was also evaluated using the *C*-statistic, which was compared using the DeLong test.

A two-sided *P*-value <.05 was considered statistically significant. All analyses were performed using IBM SPSS Statistics software version 29.0 (IBM Corp., Armonk, NY, USA) and R version 4.4.1 (R Foundation for Statistical Computing, Vienna, Austria).

## Results

### Study population

**Figure 1:**
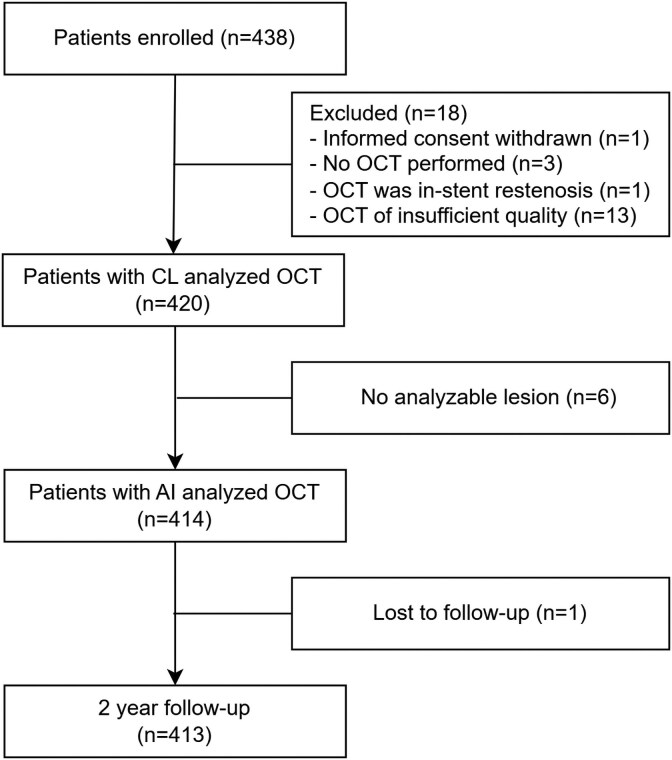
Study flow chart. AI, artificial intelligence; CL, core laboratory; OCT, optical coherence tomography

A total of 414 patients (with 488 lesions) were eligible for the present analysis (*Figure 1*). Baseline clinical and procedural characteristics are summarized in *Table 1*. The mean age was 63 ± 10 years, and 335 (80.9%) patients were male. Diabetes and hypertension were present in 14.5% and 52.9% of patients, respectively. The proportions of patients who had presented with ST-segment elevation (51.4%) and with non-ST-segment elevation (48.6%) were comparable.

**Table 1:** Baseline characteristics

Characteristic	*n*	Total (*n* = 414)
Patients	414	
Age, years		63 ± 10
Male sex		335 (80.9%)
BMI, kg/m^2^	408	27.8 ± 4.6
Smoking status	411	
Current		122 (29.7%)
Previous		125 (30.4%)
Never		164 (39.9%)
Hypertension		219 (52.9%)
Type 1 or 2 diabetes		60 (14.5%)
Hypercholesterolemia	413	152 (36.7%)
Family history of premature atherosclerosis	410	123 (30.5%)
Previous MI		63 (15.2%)
Previous PCI		62 (15.0%)
Previous CVA		8 (1.9%)
History of carotid artery disease		13 (3.1%)
History of PAD		17 (4.1%)
Index presentation		
STEMI		213 (51.4%)
NSTEMI		201 (48.6%)
GRACE score	411	117 ± 33
Cholesterol level, mmol/L		
Total	349	4.98 ± 1.36
LDL	329	3.04 ± 1.20
Triglyceride level, mmol/L	345	1.60 (1.06–2.20)
eGFR, mL/min	410	79.8 ± 19.1
CRP, mg/L	325	2.80 (1.00–5.00)
Leukocyte count, ×10^9^/L	408	9.76 ± 3.23
Lipid-lowering therapy at presentation		108 (26.1%)
Infarct-related artery		
LM		5 (1.2%)
LAD		166 (40.1%)
Cx		105 (25.4%)
RCA		155 (37.4%)
Number of target lesions		1.18 ± 0.42
Target vessel distribution		
LM		4 (1.0%)
LAD		173 (41.8%)
Cx		151 (36.5%)
RCA		121 (19.2%)

AI-TCFA, artificial intelligence-identified thin-cap fibroatheroma; BMI, body mass index; CRP, C-reactive protein; CVA, cerebrovascular accident; LAD, left anterior descending artery; Cx, circumflex artery; eGFR, estimated glomerular filtration rate; FFR, fractional flow reserve; LDL, low-density lipoprotein; LM, left main; MI, myocardial infarction; NSTEMI, non-ST-elevation myocardial infarction; PAD, peripheral artery disease; PCI, percutaneous coronary intervention; RCA, right coronary artery; STEMI, ST-elevation myocardial infarction.

Target lesions were most frequently located in the left anterior descending artery (37.9%) and the left circumflex artery (33.6%), and had a mean FFR of 0.89 ± 0.05 (*Table 2*). Based on core laboratory quantitative OCT analyses, the mean lesion length was 18.8 ± 8.8 mm. The mean minimum lumen diameter and mean minimum lumen area were 1.51 ± 0.44 mm and 2.70 ± 1.53 mm^2^, respectively.

**Table 2:** Lesion-level characteristics

Characteristic	Total (*n* = 488)
Lesion distribution	
LM	4 (0.8%)
LAD	185 (37.9%)
Cx	164 (33.6%)
RCA	135 (27.7%)
Fractional flow reserve	0.89 ± 0.05
Quantitative OCT analyses	
Lesion length, mm	18.8 ± 8.8
Minimum lumen diameter, mm	1.51 ± 0.44
Minimum lumen area, mm^2^	2.70 ± 1.53
Percentage area stenosis	62.3 ± 16.0

Cx, circumflex artery; LAD, left anterior descending artery; LM, left main stem; OCT, optical coherence tomography; RCA, right coronary artery.

### Automated artefact detection

The median percentage of excluded number of frames per lesion based on artefact detection was 0% (IQR 0–0%, 90th percentile 3.2%). Consequently, the median number of analyzable frames per lesion was 195 (136–264) compared with 201 (137–268) frames before artefact identification. A total of 6 patients had more than 50% non-analyzable frames and were excluded from further analysis.

### AI-based TCFA identification vs. core laboratory reference in target lesions

**Figure 2:**
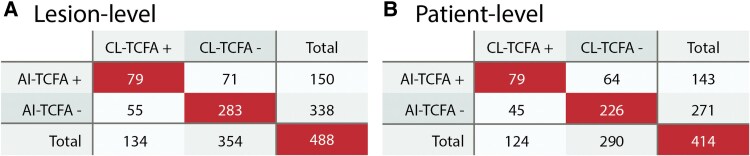
Correspondence between AI-TCFA and CL-TCFA. Contingency tables for the correspondence between AI-TCFA and CL-TCFA on a lesion-level (*A*) and patient-level (*B*). AI, artificial intelligence; AI-TCFA, artificial intelligence-identified thin-cap fibroatheroma; CL-TCFA, core laboratory-identified thin-cap fibroatheroma

AI-TCFA was present in 150 (30.7%) target lesions, while CL-TCFA was present in 134 (27.5%) target lesions. Fair to moderate agreement was observed between AI-TCFA and CL-TCFA (*κ* = 0.38, 95% CI: .28–.47) on a lesion-level. Overall, the core laboratory and the AI algorithm agreed on the presence and absence of TCFA in 79 (16.2%) and 283 (58.0%) lesions, respectively (*Figure 2A*). For lesions that qualified as CL-TCFA but not as AI-TCFA (*n* = 55), the median AI-identified minimum fibrous cap thickness on CL-TCFA-containing frames was 81 µm (IQR 71–104 µm). For lesions that qualified as AI-TCFA but not as CL-TCFA (*n* = 71), the median CL-identified minimum fibrous cap thickness within the lesion was 105 µm (IQR 78–138 µm) among 55 lesions with CL-identified lipid. The remaining 16 lesions are presented in Supplementary data online, *Figure S1*.

On a patient-level, 143 (34.5%) patients had at least one AI-TCFA in any target lesion, while 271 (65.5%) had no AI-TCFA. CL-TCFA was present in 124 (30.0%) patients and absent in 290 (70.0%) patients. The patient-wise agreement for TCFA identification was fair to moderate (*κ* = 0.40, 95% CI: .30–.49) (*Figure 2B*).

### TCFA in target lesions and clinical outcome

**Figure 3:**
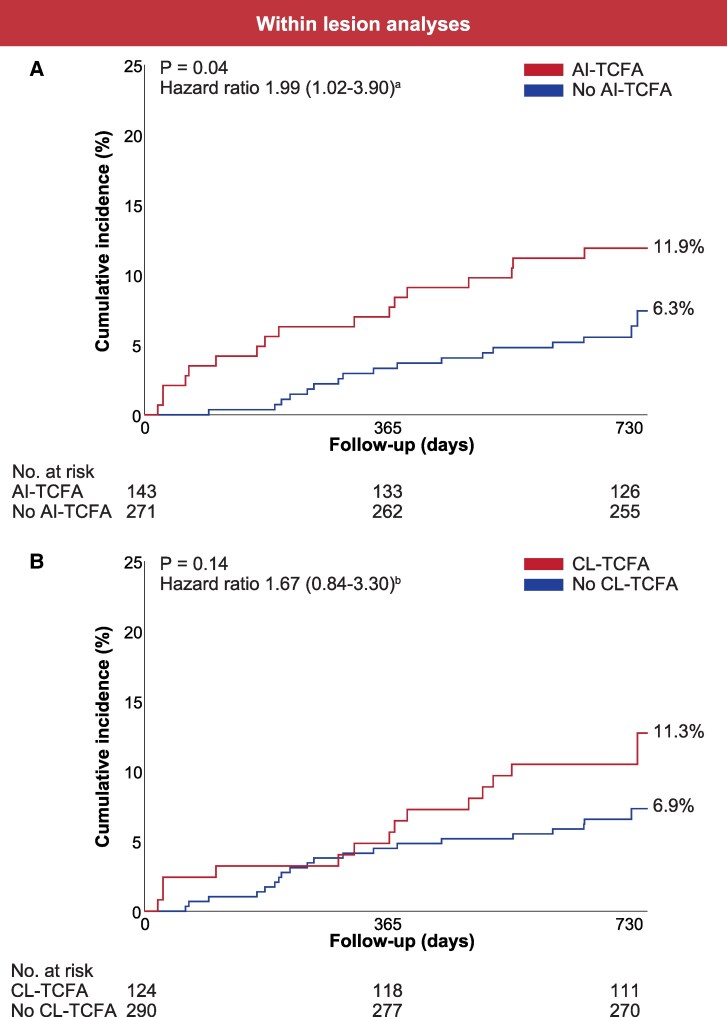

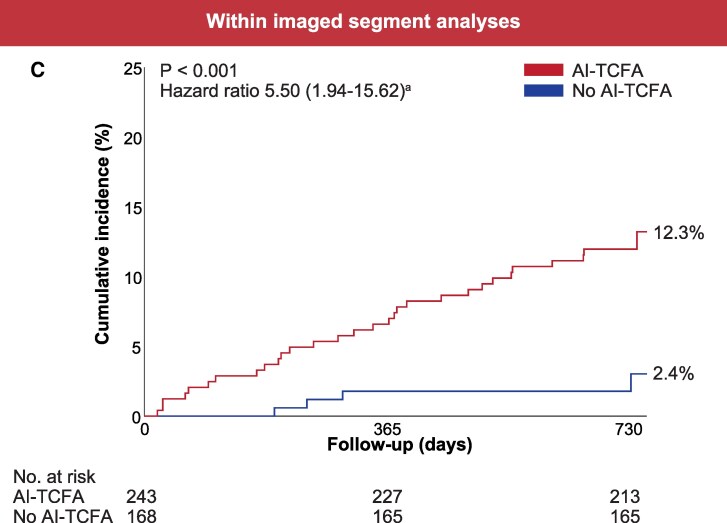
Survival curves for the primary outcome according to the presence or absence of TCFA. Survival curves for the primary composite outcome according to the presence or absence of AI-TCFA in the target lesion (*A*), CL-TCFA in the target lesion (*B*) and AI-TCFA in the complete imaged segment (*C*). AI-TCFA, artificial intelligence-identified thin cap fibroatheroma; CL-TCFA, core laboratory-identified thin cap fibroatheroma. * Patient-level comparison of AI-TCFA positive vs. AI-TCFA negative cases. ^†^ Patient-level comparison of CL-TCFA positive vs. CL-TCFA negative cases.

One patient was lost to follow-up between the first and second years of follow-up. The clinical outcome at 2-year follow-up is summarized in *Table 3*. At 2-year follow-up, the primary outcome had occurred in 34 (8.2%) patients. Compared with those identified as not having AI-TCFA, those with AI-TCFA had a 1.99 (95% CI: 1.02–3.90, 11.9% vs. 6.3%, *P* = .04) higher rate of experiencing the primary outcome (*Figure 3A*). Compared with those identified as not having CL-TCFA, those with CL-TCFA had a 1.67 (95% CI: .84–3.30, 11.3% vs. 6.9%, *P* = .14) higher rate of experiencing the primary outcome (*Figure 3B*). The C-statistic for AI-based evaluation was not significantly different from core laboratory-based analysis (0.58, 95% CI: .50–.67 vs. 0.56, 95% CI: .47–.65, *P* = .65).

**Table 3:** Clinical outcome at two-year follow-up according to the presence or absence of TCFA

Within lesion analyses (*N* = 414)				
	AI-TCFA (*n* = 143)	No AI-TCFA (*n* = 271)	*P*-value	Univariable HR (95% CI)^a^
Primary outcome	17 (11.9%)	17 (6.3%)	.04	1.99 (1.02–3.90)
Death	6 (4.2%)	8 (3.0%)	.49	1.46 (.51–4.19)
Non-fatal MI	2 (1.4%)	5 (1.8%)	.76	0.77 (.15–3.98)
Unplanned revascularization	12 (8.4%)	9 (3.3%)	.02	2.65 (1.12–6.28)

	CL-TCFA (*n* = 124)	No CL-TCFA (*n* = 290)	*P*-value	Univariable HR (95% CI)^b^
Primary outcome	14 (11.3%)	20 (6.9%)	.14	1.67 (.84–3.30)
Death	5 (4.0%)	9 (3.1%)	.62	1.32 (.44–3.93)
Non-fatal MI	4 (3.2%)	3 (1.0%)	.12	3.10 (.69–13.87)
Unplanned revascularization	9 (7.3%)	12 (4.1%)	.19	1.77 (.75–4.21)

Within imaged segment analyses (*N* = 411)				
	AI-TCFA (*n* = 243)	No AI-TCFA (*n* = 168)	*P*-value	Univariable HR (95% CI)^a^
Primary outcome	30 (12.3%)	4 (2.4%)	<.001	5.50 (1.94–15.62)
Death	13 (5.3%)	1 (0.6%)	.009	9.34 (1.22–71.39)
Non-fatal MI	6 (2.5%)	1 (0.6%)	.14	4.30 (.52–35.69)
Unplanned revascularization	18 (7.4%)	3 (1.8%)	.01	4.35 (1.28–14.78)

AI-TCFA, artificial intelligence-identified thin-cap fibroatheroma; CI, confidence interval; HR, hazard ratio; MI, myocardial infarction.

^a^Patient-level comparison of AI-TCFA positive vs. AI-TCFA negative cases.

^b^Patient-level comparison of CL-TCFA positive vs. CL-TCFA negative cases.

### TCFA across the complete imaged segment and clinical outcome

At least one complete imaged segment was analyzable for >50% in 411 patients, of whom 243 (59.1%) had AI-TCFA. Those with AI-TCFA within the complete imaged segment had a 5.50 (95% CI: 1.94–15.62, *P* < .001) higher rate of experiencing the primary outcome than those without any AI-TCFA (*Figure 3C*, *Table 3*). While non-fatal myocardial infarction occurred numerically more frequently in patients with vs. without AI-TCFA (2.5% vs. 0.6%, *P* = .14), a statistically higher incidence of death (5.3% vs. 0.6%, *P* = .009) and of unplanned revascularization (7.4% vs. 1.8%, *P* = .01) were observed in patients with AI-TCFA.

AI-based evaluation of the complete imaged segment had superior discriminatory ability compared with core laboratory-based evaluation of the target lesion only (C-statistics 0.66, 95% CI: .60–.72 vs. 0.56, 95% CI: .47–.65, *P* = .03). A numerical difference in C-statistic was observed in favour of AI-based evaluation of the complete imaged segment compared with AI-based evaluation of the target lesion only; however, without reaching statistical significance (0.66, 95% CI: .60–.72 vs. 0.58, 95% CI: .50–.67, *P* = .08).

## Discussion

This study evaluated the value of OCT-AID for prognostication of patients after myocardial infarction through the identification of TCFA. The main findings were: (i) AI-based TCFA identification using OCT was feasible, requiring exclusion of only a very small percentage of frames; (ii) the agreement between AI-TCFA and CL-TCFA was fair to moderate on both a patient level and lesion level; (iii) AI-TCFA showed a significant association with adverse cardiovascular events; (iv) incremental prognostic discriminatory value of AI was achieved by evaluating a complete OCT pullback compared with evaluation of the target lesion only. These results indicate the potential of AI for prognostication in a robust, standardized and comprehensive manner, offering an alternative to manual, non-standardized, on-site image assessment, and potentially facilitating ad-hoc interventions (Structured Graphical Abstract).

To overcome the interobserver variability in high-risk plaque identification, which is a laborious task, automated methodologies are warranted. Such methodologies would ideally allow standardisation of OCT image interpretation with accuracies comparable to dedicated core laboratories, and most importantly with an association with clinical outcome. We previously demonstrated that our deep learning-based algorithm can provide accurate full-vessel segmentation and plaque quantification.^9^ In the present study, we only found a fair to moderate correspondence between AI-TCFA and CL-TCFA, which is, however, in line with previous studies demonstrating important interobserver variability for TCFA identification.^6,7^ Furthermore, the median AI-determined minimum fibrous cap thickness on CL-TCFA-containing frames was close to the 65 µm cut-off value used to define TCFA.

Despite this limited agreement, AI-TCFA was significantly associated with the patient-level primary outcome, with discriminatory value at least comparable to core laboratory-based analyses when evaluated within the target lesion and superior discriminatory value when used to evaluate the complete imaged segment. These results are indicative of the potential of AI for prognostication. First, OCT-AID allows rapid and comprehensive evaluation of each analyzable frame, while manual image analyses are generally limited to screening of the lesion, with at best measurements in a single frame or limited number of frames. Second, the proposed methodology allows reproducible image evaluation and can thereby help bridge the gap between findings from standardized research settings and daily clinical practice. Additionally, it overcomes the limitation of manual interobserver variability. For example, the randomized PREVENT trial demonstrated that preventive treatment of high-risk plaques may be beneficial; however, the discrepancies between manual on-site image evaluation and *post hoc* core laboratory-based analyses accentuated the challenges in high-risk plaque detection.^8^ Therefore, the proposed algorithm has the potential to enable standardized, periprocedural assessment of OCT images, potentially facilitating *ad hoc* interventions for high-risk plaques. Future validation studies are required to support the findings of our study and enhance the validity of our AI-based approach. In that regard, a validation study comparing the algorithm to histology as the reference standard and a web-based reader study are currently ongoing. Ultimately, prospective clinical evaluation is warranted to evaluate the feasibility and value of an AI-based high-risk plaque identification in clinical care.

A small number of studies have previously applied AI for the same purpose. Niioka *et al*.^15^ evaluated a frame-wise classification algorithm for the presence of TCFA and found a significant association with AI-identified TCFA and both patient-level and lesion-level events.^15^ However, classification algorithms generally have lower explainability compared with semantic segmentation models such as OCT-AID of which the predictions can be compared with the corresponding pixels. Hong *et al*.^16^ defined the lipid-to-cap ratio as new measurement to identify high-risk plaques using another semantic segmentation model. Patients with an lipid-to-cap ratio >0.33 were at an almost 20-fold increased risk of patient-level non-culprit lesion events.^16^ Finally, Biccirè *et al*.^17^ obtained a lipid-spread out plot using deep learning alike those normally obtained using near infrared spectroscopy. When evaluated in the CLIMA dataset,^2^ the lipid core burden index within 4 mm was associated with a 2.5-fold increased risk of the primary CLIMA endpoint, which was below the HRs obtained using core laboratory analyses.^17^ However, core laboratory analyses were performed with different plaque characteristics, limiting direct comparison between AI and core laboratory findings. In contrast, our direct comparison between core lab and AI findings demonstrates, for the first time, that AI can be at least comparable to core laboratory-based evaluation for prognostication through high-risk plaque identification.

Intriguingly, AI-TCFA was found outside of the target lesion in ∼35% of patients without AI-TCFA in the target lesion, and an even stronger association was observed with AI-TCFA with adverse outcome when evaluating the complete imaged segment. These results indicate that plaque vulnerability is not limited to visually identifiable non-culprit lesions but that it affects the whole epicardial coronary vasculature. Remarkably, AI identified all but one patient who died during the first 2 years of follow-up, and <2% of patients without AI-TCFA required revascularisation during follow-up. This very high negative predictive value (97.6%) could be of great clinical value for prognostication after myocardial infarction. These findings again emphasizes the potential value of AI, especially considering that comprehensive, manual image evaluation of a complete imaged segment is practically impossible on-site.

Importantly, AI-TCFA in the present study was defined according to standard criteria based on single-point measurements. To reduce the risk of false positive TCFA diagnoses due to single measurements, we applied a threshold of three frames meeting the TCFA criteria before a lesion would qualify as TCFA. Such threshold mimics human interpretation, as it is unlikely that human observers would classify a lesion as TCFA-positive if only one frame would meet the defined criteria while the others do not. Whether more comprehensive plaque analyses enabled through the application of AI and that may be impossible to perform manually,^18^ provide superior predictive value remains speculative.

### Limitations

This study was conducted on the data from a single prospective observational study, which may limit the generalizability of the findings. Furthermore, the PECTUS-obs study was not specifically designed to test superiority of AI-based OCT analysis compared with core laboratory-based analysis. Future validation studies are therefore warranted. Furthermore, the AI-based OCT analyses were performed offline. Therefore, it remains to be prospectively investigated how the proposed algorithm would perform and would be perceived when adopted directly on-site after OCT image acquisition. This is of particular importance when focal treatment of high-risk plaques would be performed. Additionally, core laboratory analyses were only available for the target lesion, precluding comparison between AI and core laboratory analyses in the complete imaged segment. Finally, PECTUS-obs was designed to address the high-risk patient hypothesis, and was not powered to demonstrate differences in lesion-level events. Likewise, the study was not powered to evaluate differences in individual components of the composite outcome. The final 5-year follow-up results may provide additional insights in that regard.

## Conclusions

AI-based OCT image analysis allows standardized identification of patients at increased risk of adverse cardiovascular outcome, offering an alternative to manual image analysis. Furthermore, AI-assisted evaluation of complete imaged segments provides more prognostic impact than evaluation of the target lesion only.

## Supplementary Material

ehaf595_Supplementary_Data

## Declarations

### Data Availability

The data underlying this article are available from the corresponding author upon reasonable request. The code and annotated data underlying this manuscript cannot be made publicly available due to funding source restrictions.

### Pre-registered Clinical Trial Number

NCT03857971.
