# Supplementary material for: Artificial intelligence-based identification of thin-cap fibroatheromas and clinical outcomes: the PECTUS-AI study
Source: Eur Heart J. 2025 Sep 1;46(46):5032–41. doi: 10.1093/eurheartj/ehaf595 (PMC12682388; doi:10.1093/eurheartj/ehaf595)
Supplement: ehaf595_Supplementary_Data [file ehaf595_supplementary_data.docx]

**Supplemental appendix**

Artificial intelligence-based identification of thin-cap fibroatheromas in relation to clinical outcome:
the PECTUS-AI study.

Content

[Supplemental Methods 3](#_Toc205367313)

[Method S1 Inclusion and exclusion criteria 3](#_Toc205367314)

[Method S2 Multiclass semantic segmentation algorithm 4](#_Toc205367315)

[Method S3 Stratified training/testing splitting scheme 5](#_Toc205367316)

[Method S4 Frame-wise classification and pixel-wise segmentation performance across training folds 6](#_Toc205367317)

[Supplemental Results 7](#_Toc205367318)

[Figure S1 Discrepancies 7](#_Toc205367319)

[Supplemental References 8](#_Toc205367320)

# Supplemental Methods

## Method S1 Inclusion and exclusion criteria

| **Inclusion criteria** | **Exclusion criteria** |
| --- | --- |
| Clinical | Clinical |
| Age ≥18 years Hospitalization with a STEMI or NSTEMI for which patient is subjected to invasive coronary angiography (within the last 6 weeks) | Pregnancy Hemodynamic instability, respiratory failure or Killip class ≥3 at time of inclusion Previous CABG Indication for revascularization by CABG Estimated life expectancy <3 years |
| Angiographical | Angiographical |
| Patient has ≥1 non-culprit, target lesion(s) with following additional characteristics: - Lesion has visual stenosis of 30-90% - Lesion is non-obstructive (FFR >0.80) - Lesion is not in-stent restenosis | Anatomy of target lesion(s) is unsuitable for OCT catheter crossing or imaging (aorta-ostial lesions, too small diameter segment, severe calcifications, chronic total occlusion, distal lesions prohibiting OCT imaging) |

CABG coronary artery bypass grafting; FFR fractional flow reserve; NSTEMI non-ST-segment elevation myocardial infarction; OCT optical coherence tomography; STEMI ST-segment elevation myocardial infarction.

## Method S2 Multiclass semantic segmentation algorithm

An updated version of the multiclass semantic segmentation algorithm (OCT-AID)^1^, previously developed by our group, was developed for pixelwise labeling of the OCT pullbacks. The updated model was based on the nnU-Net version 2, introducing a residual encoder U-Net architecture.^2^ The training hyperparameters were as follows:

| **Hyperparameter** | **Value** |
| --- | --- |
| Image coordinates | Cartesian |
| Input image size | 704 x 704 x 7 in greyscale |
| Output image size | 704 x 704 x 1 |
| Batch size | 5 |
| Learning rate | 0.01 |
| Learning rate scheduler | Polynomial learning rate decay |
| Optimizer | Stochastic gradient descent |
| Epochs | 1000 |
| Loss function | Equally weighted Dice and cross-entropy loss |
| Weight decay coefficient | 0.00003 |
| Number of learnable parameters | 126M |

The model predictions were post-processed using connected component analysis to remove small isolated pixel areas. Additional details on the algorithm architecture and development are described elsewhere.^1^

## Method S3 Stratified training/testing splitting scheme


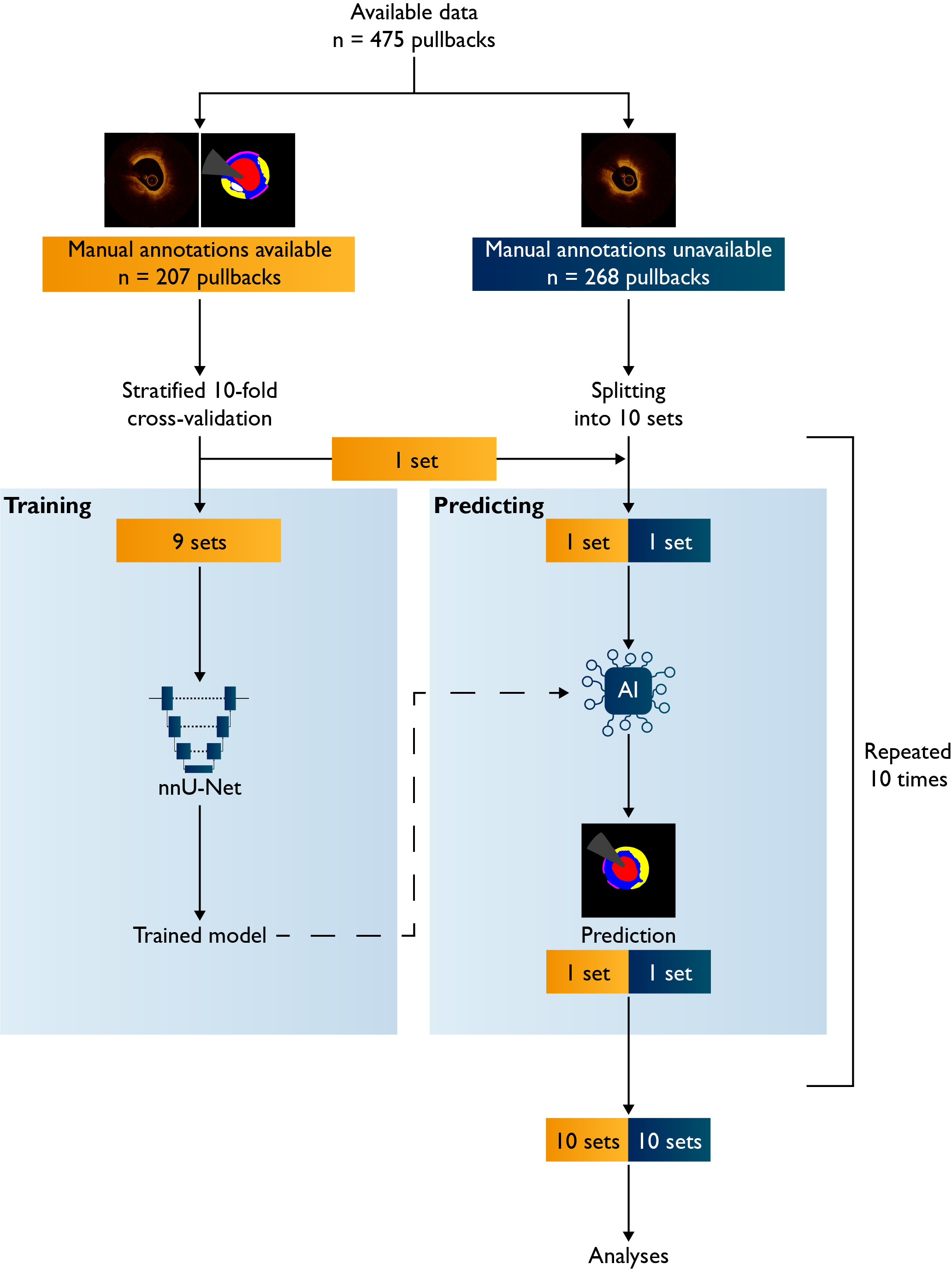


AI Artificial Intelligence

## Method S4 Frame-wise classification and pixel-wise segmentation performance across training folds

|  | **Media** | | **Lipid** | | **Calcium** | | **Side branch** | | **Plaque rupture** | | **Thrombus** | |
| --- | --- | --- | --- | --- | --- | --- | --- | --- | --- | --- | --- | --- |
|  | **Sensitivity** | **Specificity** | **Sensitivity** | **Specificity** | **Sensitivity** | **Specificity** | **Sensitivity** | **Specificity** | **Sensitivity** | **Specificity** | **Sensitivity** | **Specificity** |
| 1 | 98 (95-99) | 70 (40-89) | 95 (91-97) | 78 (70-84) | 93 (85-96) | 96 (92-98) | 82 (69-91) | 96 (92-98) | 62 (42-78) | 96 (93-98) | 81 (68-90) | 98 (96-99) |
| 2 | 97 (94-98) | 80 (55-93) | 95 (91-97) | 88 (81-92) | 88 (80-93) | 92 (87-95) | 83 (71-90) | 95 (92-97) | 74 (51-88) | 97 (95-99) | 92 (78-97) | 97 (94-98) |
| 3 | 96 (93-98) | 97 (83-99) | 93 (89-96) | 88 (82-92) | 85 (77-91) | 94 (90-96) | 86 (75-93) | 96 (94-98) | 83 (65-92) | 99 (97-99) | 90 (75-97) | 99 (97-99) |
| 4 | 100 (98-100) | 83 (55-95) | 89 (82-93) | 91 (85-94) | 91 (83-96) | 93 (89-96) | 82 (69-90) | 98 (96-99) | 67 (35-88) | 98 (95-99) | 85 (58-96) | 98 (96-99) |
| 5 | 99 (97-100) | 65 (43-82) | 94 (90-97) | 89 (83-93) | 85 (79-90) | 96 (92-98) | 88 (78-94) | 99 (97-99) | 58 (42-73) | 97 (95-99) | 94 (82-98) | 96 (93-98) |
| 6 | 97 (94-99) | 60 (31-82) | 95 (90-97) | 84 (77-90) | 94 (88-97) | 95 (91-97) | 85 (74-92) | 96 (93-98) | 75 (47-91) | 98 (95-99) | 94 (81-98) | 100 (98-100) |
| 7 | 98 (96-99) | 94 (73-99) | 93 (88-96) | 80 (72-85) | 86 (78-91) | 89 (84-92) | 83 (71-90) | 96 (93-98) | 93 (70-99) | 97 (95-99) | 93 (70-99) | 98 (96-99) |
| 8 | 97 (94-98) | 82 (52-95) | 97 (93-99) | 84 (77-89) | 91 (84-95) | 95 (91-97) | 84 (71-92) | 94 (91-97) | 78 (45-94) | 96 (93-98) | 47 (25-70) | 99 (97-100) |
| 9 | 100 (98-100) | 90 (70-97) | 95 (91-98) | 88 (81-93) | 85 (76-91) | 96 (92-98) | 80 (65-89) | 97 (94-99) | 87 (70-95) | 100 (98-100) | 85 (69-93) | 98 (96-99) |
| 10 | 99 (96-100) | 78 (45-94) | 94 (89-97) | 82 (75-88) | 95 (87-98) | 93 (88-96) | 78 (62-88) | 99 (96-100) | 82 (52-95) | 98 (95-99) | 91 (72-97) | 98 (95-99) |
| Mean | 98 | 80 | 94 | 85 | 89 | 94 | 83 | 97 | 76 | 98 | 85 | 98 |

Sensitivity and specificity per class reported as value (interquartile range).

|  | **Lumen** | **Guidewire** | **Intima** | **Media** | **Lipid** | **Calcium** | **Side branch** | **Plaque rupture** | **Thrombus** |
| --- | --- | --- | --- | --- | --- | --- | --- | --- | --- |
| 1 | 0.99 ± 0.03 | 0.93 ± 0.05 | 0.91 ± 0.06 | 0.81 ± 0.18 | 0.77 ± 0.14 | 0.81 ± 0.19 | 0.75 ± 0.31 | 0.50 ± 0.31 | 0.65 ± 0.30 |
| 2 | 0.99 ± 0.02 | 0.94 ± 0.04 | 0.90 ± 0.07 | 0.81 ± 0.15 | 0.73 ± 0.18 | 0.78 ± 0.26 | 0.78 ± 0.21 | 0.58 ± 0.35 | 0.67 ± 0.27 |
| 3 | 0.99 ± 0.01 | 0.93 ± 0.08 | 0.90 ± 0.07 | 0.80 ± 0.17 | 0.74 ± 0.17 | 0.81 ± 0.21 | 0.83 ± 0.19 | 0.74 ± 0.25 | 0.65 ± 0.27 |
| 4 | 0.99 ± 0.02 | 0.94 ± 0.06 | 0.91 ± 0.07 | 0.84 ± 0.13 | 0.77 ± 0.17 | 0.82 ± 0.20 | 0.80 ± 0.23 | 0.58 ± 0.18 | 0.41 ± 0.32 |
| 5 | 0.99 ± 0.03 | 0.93 ± 0.06 | 0.91 ± 0.06 | 0.82 ± 0.15 | 0.72 ± 0.18 | 0.74 ± 0.25 | 0.82 ± 0.22 | 0.58 ± 0.30 | 0.68 ± 0.31 |
| 6 | 0.99 ± 0.03 | 0.93 ± 0.06 | 0.89 ± 0.08 | 0.79 ± 0.16 | 0.74 ± 0.15 | 0.78 ± 0.23 | 0.80 ± 0.22 | 0.51 ± 0.26 | 0.64 ± 0.30 |
| 7 | 0.99 ± 0.01 | 0.93 ± 0.07 | 0.90 ± 0.07 | 0.81 ± 0.14 | 0.72 ± 0.16 | 0.83 ± 0.18 | 0.78 ± 0.24 | 0.64 ± 0.28 | 0.60 ± 0.30 |
| 8 | 0.99 ± 0.01 | 0.94 ± 0.04 | 0.89 ± 0.08 | 0.83 ± 0.15 | 0.78 ± 0.16 | 0.79 ± 0.23 | 0.82 ± 0.21 | 0.64 ± 0.31 | 0.62 ± 0.21 |
| 9 | 0.99 ± 0.02 | 0.94 ± 0.05 | 0.91 ± 0.06 | 0.84 ± 0.15 | 0.77 ± 0.14 | 0.80 ± 0.17 | 0.83 ± 0.20 | 0.64 ± 0.19 | 0.71 ± 0.21 |
| 10 | 0.99 ± 0.02 | 0.94 ± 0.06 | 0.90 ± 0.07 | 0.83 ± 0.14 | 0.73 ± 0.17 | 0.74 ± 0.23 | 0.83 ± 0.19 | 0.60 ± 0.26 | 0.62 ± 0.28 |
| Mean | 0.99 | 0.94 | 0.90 | 0.82 | 0.75 | 0.79 | 0.80 | 0.60 | 0.63 |

Mean per-frame Dice score ± standard deviation calculated across true-positive frames.

# Supplemental Results

## Figure S1 Discrepancies


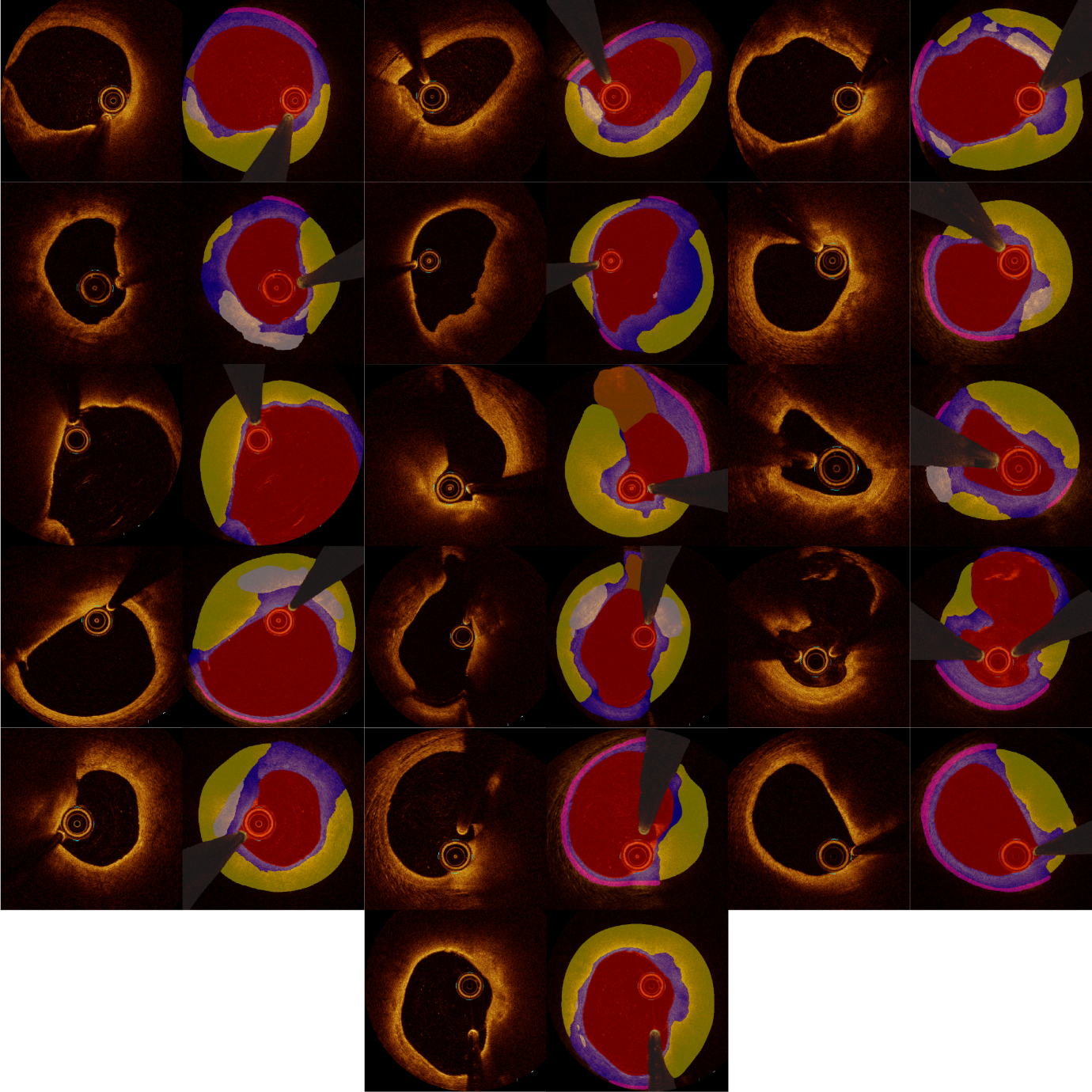


OCT images (left) with artificial intelligence-based predictions (right) of cases in which the algorithm identified a thin-cap fibroatheroma but the core laboratory did not. Grey represents guidewire artefact, red represents lumen, orange represents side branch, blue represents intima, pink represents media, yellow represents lipid and white represents calcium.

# Supplemental References

1. Volleberg RHJA, van der Waerden RGA, Luttikholt TJ, et al. Comprehensive full-vessel segmentation and volumetric plaque quantification for intracoronary optical coherence tomography using deep learning. *European Heart Journal - Digital Health* 2025.

2. Isensee F, Wald T, Ulrich C, et al. nnU-Net Revisited: A Call for Rigorous Validation in 3D Medical Image Segmentation. 2024; Cham: Springer Nature Switzerland; 2024. p. 488-98.

3. Cancian P, Saitta S, Gu X, et al. Attenuation artifact detection and severity classification in coronary OCT using mixed image representations. *SPIE Medical Imaging, Image Processing* 2025.
